# The role of dulaglutide in the treatment of alcohol use disorder: a case report

**DOI:** 10.3389/fpsyt.2025.1420316

**Published:** 2025-05-01

**Authors:** Olivia Hill, Sarah Hughes, Aakanksha Singh, Michael Ang-Rabanes, Raja Mogallapu

**Affiliations:** Department of Behavioral Medicine and Psychiatry, School of Medicine, West Virginia University, Morgantown, WV, United States

**Keywords:** dulaglutide, alcohol use disorder (AUD), substance and alcohol use, GLP-1 receptor agonist, semaglutide

## Abstract

Glucagon-like peptide 1 (GLP-1) receptor agonists, medications commonly employed in the treatment of type 2 diabetes mellitus, have illustrated several additional benefits, including weight loss and potentially reduce addictive cravings. Several studies have indicated that GLP-1 receptor agonists may be effective in treating Alcohol Use Disorder (AUD), for which current pharmacologic therapies are often inadequate. Proposed mechanisms include modulation of dopaminergic transmission and reduced gastric emptying, both of which reduce alcohol craving and tolerance. This case report discusses dulaglutide’s ability to reduce alcohol consumption. During a visit to an outpatient behavioral health clinic, a 44-year-old male was evaluated for weight loss. His medical history revealed a BMI of 41.8, hypertension, major depressive disorder, and pre-diabetes. The individual also reported the consumption of approximately ninety beers per month and was in the pre-contemplation phase of change. As part of the treatment plan, the patient was prescribed dulaglutide to manage pre-diabetes and facilitate weight loss. During subsequent appointments, the individual not only experienced weight loss but also noted a substantial reduction in alcohol cravings and consumption. However, following a lapse in insurance coverage the following year, the individual had to discontinue his dulaglutide, resulting in a return to previous drinking patterns. Future research should focus on confirming existing animal study results in humans, with the hope that GLP-1 receptor agonists can become a mainstay treatment for AUD.

## Introduction

Alcohol use disorder (AUD) affects 29.5 million individuals greater than 12 years old and is one of the top causes of avoidable deaths, causing approximately 178,000 deaths annually (1). Additionally, its costs are devastating, both on an individual and national level, contributing to nearly 12% of healthcare costs in the US (2). Despite its impact, a small percentage (less than 10% of adults with AUD) receive treatment (1).

The Alcohol Use Disorders Identification Test (AUDIT) is a screening tool developed by the World Health Organization (WHO) to help individuals recognize potential alcohol-related issues (3). It offers a standardized approach for assessing alcohol consumption and potential alcohol-related risks and is widely used by healthcare professionals around the world (3). The AUDIT quantifies a patient’s frequency and amount of alcohol consumed, as well as the way it affects a patient’s social and mental health (3). Patients often conceal their alcohol use and may be hesitant to seek assistance, so it is crucial for physicians to promptly screen for alcohol use disorder using screening tests to mitigate the associated risks (4).

GLP-1 receptor agonists, developed initially as diabetes medications, have gained popularity due to their ability to suppress appetite and reduce the speed of gastric emptying, resulting in weight loss (5). Additionally, these drugs have been found to potentially mitigate substance use disorders, including AUD, due to their effects on the brain-gut axis and dopaminergic centers of the brain in pre-clinical trials (5). However, research has shown that there may be more complicated mechanisms not restricted to dopaminergic signaling and the brain-gut axis, such as insulin receptors in the brain, single nucleotide polymorphisms (SNP), and neuroplasticity (6–8). These mechanisms indicate that they have the potential to become a very useful treatment in more diseases than just diabetes and while these drugs are expensive and may increase healthcare costs in the short run, it may reduce costs in the long run.

This case report serves to augment the existing body of evidence supporting the favorable impact of GLP-1 receptor agonists, not only on diabetes and weight management but also on AUD. The utilization of laboratory tests, dulaglutide titration, counseling, regular follow-ups, and periodic AUDIT screening led to notable advancements in our patient’s weight loss and effectively addressed his AUD.

## Case presentation

**Figure 1:**
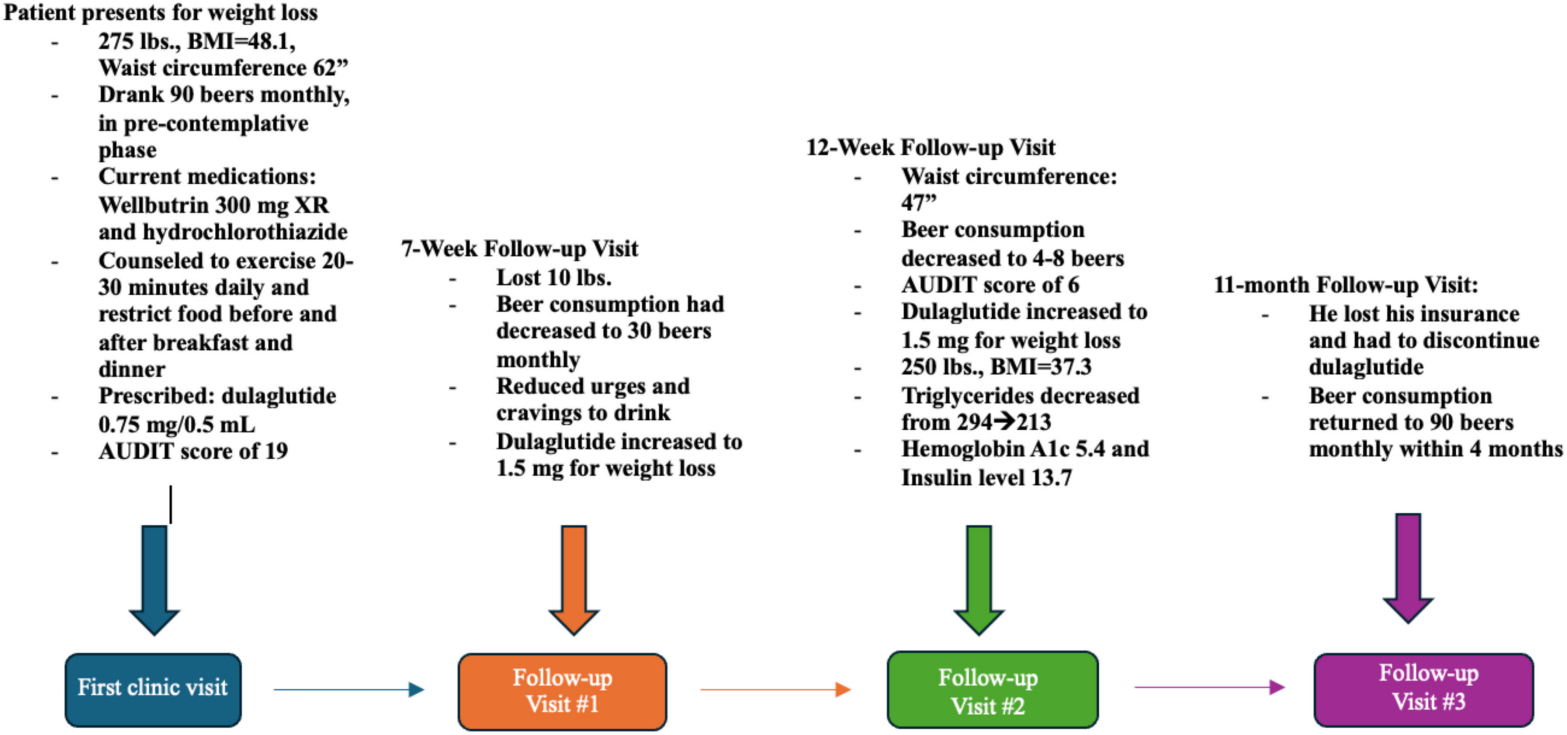
Patient visit and treatment timeline.

**Figure 2:**
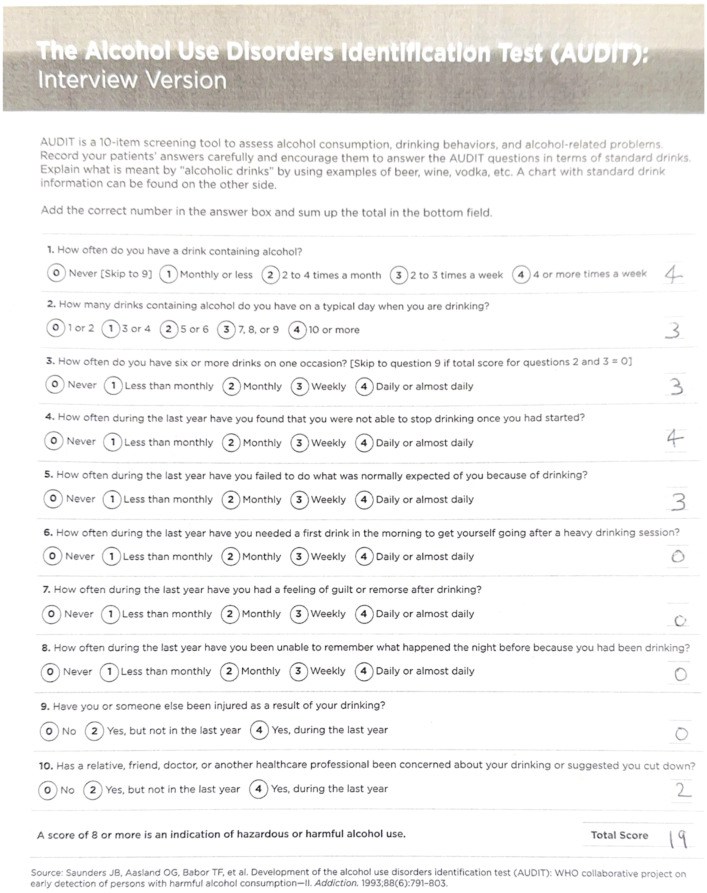
The patient’s first AUDIT score, a total of 19 (the maximum score is 40), was a positive screening result for AUD (9).

**Figure 3:**
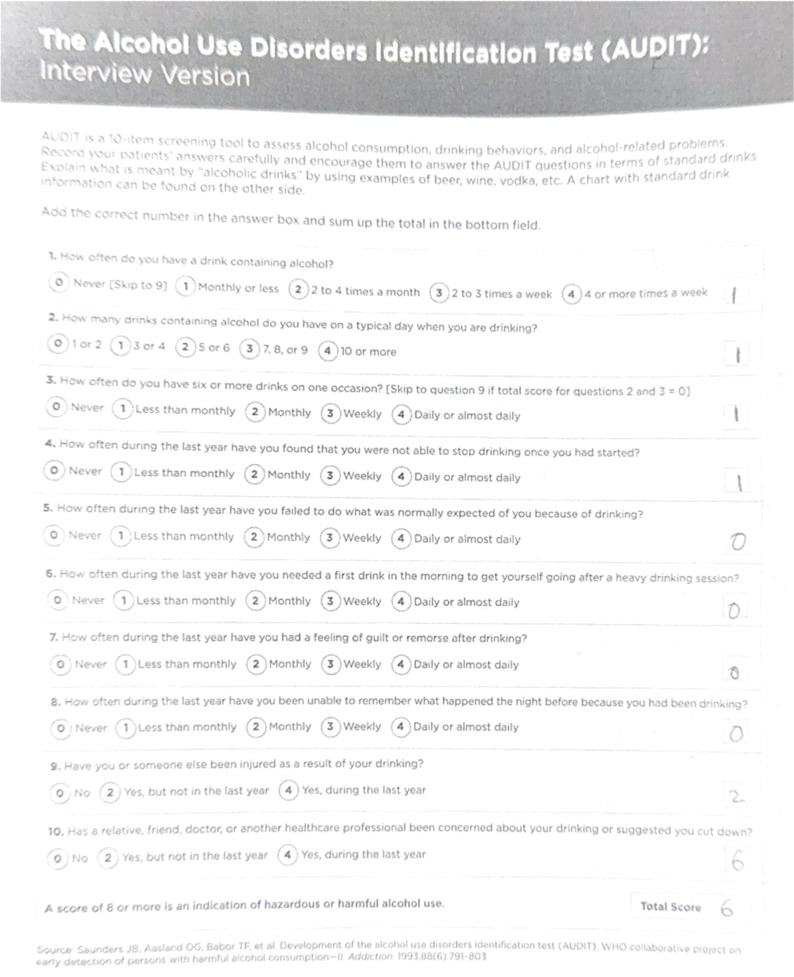
Patient’s second AUDIT score of 6 just 12 weeks later was a great improvement, and was below the threshold for a positive AUD screening value (9).

A 44-year-old male with a history of prediabetes and obesity presented at an outpatient behavioral health clinic to seek assistance with weight management and lifestyle improvement. During his visit, he weighed 275 pounds, a body mass index of 41.8 and a weight circumference of 62 inches (**Figures 1**–**3**; **Table 1**). The patient had a family history of obesity and been suffering from such since a back surgery. The patient’s previous weight loss treatment plan involved the use of phentermine, topiramate, and Wellbutrin. Wellbutrin improved his motivation and self-esteem. However, it and the phentermine did not significantly impact his weight, and he had discontinued the phentermine prior to this behavioral health clinic visit. He also had discontinued the topiramate approximately 2 days after starting it due to a metallic taste and word-finding difficulty. Other than decreased motivation and self-esteem, the patient denied any symptoms of depression. The individual also had a documented history of hypertension and was taking hydrochlorothiazide. He was prediabetic, with a hemoglobin A1c level of 5.8%, and acknowledged a diet consisting of processed foods and low levels of physical activity.

**Table 1:** Below shows the patient’s alcohol use, anthropometry, and labs with each visit.

Visit #, time since first visit	AUDIT score^1^	# Beers per month	Weight (pounds)	BMI (kg/m^2^)	Waist circumference (inches)	Insulin (mIU/mL)	Triglycerides
**First clinic visit, 0 days**	19	90	275	41.8	62	27.4	294
**Follow Up Visit #1, 56 days after**	n/a	30	265	40.3	50	n/a	n/a
**Follow Up Visit #2, 84 days after (labs taken 112 days after)**	6	4-8	250	37.3	47	13.7	227
**Follow Up Visit #3, 334 days after**	N/A	90-100	278	42	64	58.9	354

^1^The Alcohol Use Disorders Identification Test (AUDIT) is a screening tool developed by the World Health Organization (WHO) to help individuals recognize potential alcohol-related issues (3).

When questioned about substance use, he mentioned drinking approximately 90 beers per month and was in the pre-contemplation phase regarding his alcohol use. He had never been treated for AUD. The patient was screened for AUD with the AUDIT, the Alcohol Use Disorder Identification Test. This 10-question screening tool aids physicians in understanding the severity of a patient’s alcohol use disorder and supports them in choosing the most appropriate treatment (3). The patient’s AUDIT score was 19, suggesting moderate-severe alcohol use disorder (3) (**Figures 2**, **3**). The patient was warned about the dangers of alcohol withdrawal seizures and Wellbutrin and the decision was made that the benefit far outweighed the risk in his situation.

A preliminary laboratory study revealed that his insulin level was 27.4 mIU/mL, his HDL was 43, his triglycerides were 294, and his VLDL was 48. Several weight loss options were discussed, and the patient selected a weekly 0.25mg subcutaneous semaglutide injection. However, due to insurance issues, the initial prescription of semaglutide was changed to 0.75 mg of dulaglutide. The patient received behavioral counseling on nutrition and lifestyle changes, including the importance of daily exercise for 20-30 minutes, healthy eating habits, and food sequencing. The decision was also made to continue to Wellbutrin for his mood.

In his first follow-up visit seven weeks later, he reported no adverse effects from the medication and had lost ten pounds. He reported that his drinking habits had changed, and he was consuming only 30 beers per month. The patient reported feeling full after drinking and had reduced urges and cravings to drink. The patient also reported that he still was having binge eating episodes and binge drinking at times, especially in the two days prior to his next injection. During this visit, the patient agreed to increase his dulaglutide dose to 1.5 mg. He stated that he was trying to follow a healthy diet but had only minimally changed his exercise. A follow-up appointment was scheduled 5 weeks later.

In the second follow-up visit, the patient’s waist circumference was 47 inches, and he reported consuming four to eight beers per month, resulting in a new AUDIT score of 6. This was considered low-risk alcohol consumption (3). His weight had decreased to 250 pounds, with a body mass index of 37.3. His repeated laboratory tests revealed a decrease in triglyceride levels from 294 to 213, with no other significant changes observed in his lipid panel. Additionally, his HbA1c had decreased to 5.4 with an insulin level of 13.7. He denied any cravings for food or alcohol during this visit and wanted to continue the same dose of dulaglutide. The patient also reported dietary improvements but still had not improved his exercise regimen. The importance of exercise was again discussed in this visit.

In the subsequent months, the patient retired from his job due to a back injury exacerbated by physical strain. Unfortunately, the loss of health insurance made it unaffordable for him to continue using dulaglutide; however, he was able to maintain his other prescribed medications. Two weeks after discontinuing dulaglutide, he began drinking again. Within four months, his consumption escalated back to his previous average of 90 beers per month.

His third follow-up appointment took place 11 months after the initial consultation. Although it was recommended that he be seen three months earlier, he opted to cancel this visit due to undergoing back surgery. Given that he could not utilize a GLP-1 receptor agonist for his alcohol use disorder (AUD) and that his current insurance did not cover the GLP-1 receptor agonist for weight management, he attempted treatment with naltrexone. However, he had compliance issues due to adverse side effects.

The patient and his family have conveyed that he experienced a significant reduction in alcohol consumption and effective weight management while on a GLP-1 receptor agonist, which in turn fostered improvements in his family relationships. Additionally, his wife reported that he was very compliant with the weekly GLP-1 receptor agonist injections, as she closely monitored his adherence to the treatment regimen. On the contrary, he was non-compliant with naltrexone, and no significant changes were noticed in his drinking pattern with this medication.

## Discussion

Less than one-tenth of individuals with AUD received treatment in 2023, in part due to lack of effective screening and physician experience in treating AUD (1, 4). Disulfiram, acamprosate, and naltrexone are the primary pharmacological assets for AUD, but sometimes lack effectiveness (4). For example, the hepatic metabolism of naltrexone can be contraindicated for those with existing liver disease which is commonly associated with AUD (10). Additionally, patients with concurrent opioid use are also unable to use naltrexone for AUD due to precipitation of withdrawal, and acamprosate cannot be used in those with severe renal disease (4, 11). Disulfiram has very undesirable side effects, and patients often not maintain its use (4).

The left insula, hypothalamus, and orbitofrontal cortex are involved with reward, food anticipation and satiety, and GLP-1 receptors are found in these areas (5). Studies have also shown that GLP-1 decreases extracellular dopamine release and increases dopamine turnover, which decreases the reward aspect of food and alcohol (12). Multiple studies on mice have found that treatment with Exendin-4, a GLP-1 receptor agonist, reduced alcohol consumption by altering dopamine pathways (5, 12–15). In one of those studies, subjects did not have significant relapse following treatment cessation (16). It is also worth mentioning that liraglutide, which is marketed under the brand name ‘Victoza,’ has been shown to decrease alcohol consumption in rats by impacting the mesolimbic dopamine pathways (17).

AUD is associated with neuroplastic changes in the brain (6). Studies have shown that in those with AUD, networks affecting reward and self-control such as the striatum and orbitofrontal cortex have decreased synchronous activity, which is correlated with years of heavy alcohol consumption (6). Further, when those with AUD abstain from alcohol, their brain remains altered, leaving them more susceptible to relapsing due to mental and physical stress (18). Since GLP-1 receptors have been found to be involved with neural pathways, including the striatum, it is reasonable to consider if GLP-1 receptor agonists can affect the neuroplastic changes associated with AUD (6, 19). Our patient relapsed quickly after stopping his dulaglutide, leading us to believe that either there was not enough time for the neuroplastic changes to be affected, or it is not affecting these areas in a way that allows for remodeling.

SNPs in the GLP-1 receptor genes have clinically significant differences. One study showed that humans and mice with a GLP1R 168Ser non-wild type allele had a higher intravenous alcohol intake, as well as an increased signal intensity in the globus pallidus during a reward-seeking task (7). Similarly, mice with less expression of the 168Ser wild-type allele responded less to GLP-1 receptor agonists (7). While the current evidence is limited, the existing data on SNPs in the GLP-1 receptor genes and their role in alcohol and other substance use disorders shows promise and warrants future research.

Human studies on GLP-1 receptor agonists as a potential AUD treatment are limited, but more literature is starting to address this. A study from 2023 found that individuals taking semaglutide and tirzepatide self-reported significantly lower overall alcohol consumption and cravings (20). An observational study from January 2006-December 2023 found that of the 227, 866 individuals with AUD and comborbid obesity or type 2 diabetes studied, semaglutide and liraglutide were associated with a significantly lower risk of AUD hospitalization than those taking well-established AUD treatments (21) A systemic review from November 2024 showed that there was a significant reduction in substance use disorders in the 630 subjects prescribed either exenatide or dulaglutide for substance use disorder (22). A case series on 6 patients with positive AUD screenings significantly improved their AUD symptoms while using semaglutide for weight loss (23). Contrarily, a randomized, placebo-controlled clinical trial using exenatide once weekly did not reduce the number of heavy drinking days in 127 patients; however, the authors stated that the study population had drinking severity lower than other trials (24). Thus, the evidence for GLP-1 receptor agonists is increasing and overall promising, but the evidence for dulaglutide is explicitly smaller and requires more research.

Binge eating and substance use disorders have similarities in their pathophysiology (25). One study found that in females, excess alcohol use correlated with binge eating (25). GLP-1 receptor agonists are also utilized for weight loss, even in those without diabetes (5). These drugs both slow gastric emptying and influence receptors in reward-seeking areas in the CNS, and both may have aided in the patient’s improved diet (5).

Insulin plays a significant role in regulating blood sugar levels and controlling glucose and lipid metabolism (8). Insulin resistance in the brain can also lead to an increase in alcohol intake and addiction (13). In the hypothalamus, insulin resistance can affect the brain’s reward circuitry by decreasing dopamine levels in the striatum, which leads to an increased desire for alcohol and causes communication changes between brain neurons (8, 13). Our patient had a significant decrease in insulin levels after 12 weeks of treatment, which likely lowered his insulin resistance and may have contributed to his reduced alcohol cravings (13).

It is important to note that the stomach and duodenum play critical roles in alcohol metabolism and absorption (26). The stomach primarily metabolizes ethanol to acetaldehyde locally, while the duodenum is responsible for the rapid absorption of alcohol into the bloodstream (26). The delayed gastric emptying from the stomach to the duodenum following the administration of GLP-1 receptor agonists may lead to higher levels of acetaldehyde and may alter or blunt the rise in blood alcohol levels (BAC) (26). This change in the alcohol absorption curve significantly diminishes its effects and thus abuse potential, which could potentially explain the observed decrease in AUDIT scores and weekly alcohol consumption in our patient (26).

The patient presented with both weight gain and excess alcohol use. Despite the limitation of his insurance only covering dulaglutide and not semaglutide, he achieved remarkable results with his weight loss and drinking habits. The return of his cravings due to insurance issues underlines the pivotal role of insurance in patient care, as well as the need for further research on preventing rebound effects following discontinuation. Quitting his job likely acted as a social stressor and may have contributed to his relapse, but had the patient been able to stay on the dulaglutide during this stressful period, it may have helped prevent such a relapse.

## Conclusion

This case report demonstrates a case of weight loss and significant alcohol use reduction in the setting of GLP-1 receptor agonist use, drugs originally used for diabetes. There has been growing literature on the benefits of GLP-1 receptor agonists on weight loss and AUD. The widespread effects of GLP-1 in the CNS, especially on the mesolimbic pathway, and the GI tract work together to reduce cravings and urges for both food and substances. Our patient’s case of an approximately 93% reduction in alcohol consumption over only 12 weeks provides additional support for a new and potentially superior pharmacological treatment of AUD. While the results of this case report are promising, more extensive studies with a large number of participants and extended follow-up periods are needed to confirm these findings and establish the efficacy of GLP-1 receptor agonists as a treatment for AUD. Additionally, more research is required to understand the underlying mechanisms of GLP-1 receptor agonists in reducing cravings and urges for both food and alcohol.

## Data Availability

The original contributions presented in the study are included in the article/supplementary material. Further inquiries can be directed to the corresponding author.
